# Genetic and epigenetic regulators of retinal Müller glial cell reprogramming

**DOI:** 10.1016/j.aopr.2023.05.004

**Published:** 2023-06-10

**Authors:** Xueqi Xiao, Zhiyong Liao, Jian Zou

**Affiliations:** a Zhejiang Provincial Key Laboratory for Water Environment and Marine Biological Resources Protection, College of Life and Environmental Science, Wenzhou University, Wenzhou, China; b Department of Ophthalmology, The Second Affiliated Hospital of Zhejiang University School of Medicine, Hangzhou, China; c The Institute of Translational Medicine, Zhejiang University, Hangzhou, China

**Keywords:** Müller glial cell reprogramming, Genetic regulation, Epigenetic modification, retinal neuron regeneration

## Abstract

**Background:**

Retinal diseases characterized with irreversible loss of retinal nerve cells, such as optic atrophy and retinal degeneration, are the main causes of blindness. Current treatments for these diseases are very limited. An emerging treatment strategy is to induce the reprogramming of Müller glial cells to generate new retinal nerve cells, which could potentially restore vision.

**Main text:**

Müller glial cells are the predominant glial cells in retinae and play multiple roles to maintain retinal homeostasis. In lower vertebrates, such as in zebrafish, Müller glial cells can undergo cell reprogramming to regenerate new retinal neurons in response to various damage factors, while in mammals, this ability is limited. Interestingly, with proper treatments, Müller glial cells can display the potential for regeneration of retinal neurons in mammalian retinae. Recent studies have revealed that dozens of genetic and epigenetic regulators play a vital role in inducing the reprogramming of Müller glial cells in vivo. This review summarizes these critical regulators for Müller glial cell reprogramming and highlights their differences between zebrafish and mammals.

**Conclusions:**

A number of factors have been identified as the important regulators in Müller glial cell reprogramming. The early response of Müller glial cells upon acute retinal injury, such as the regulation in the exit from quiescent state, the initiation of reactive gliosis, and the re-entry of cell cycle of Müller glial cells, displays significant difference between mouse and zebrafish, which may be mediated by the diverse regulation of Notch and TGFβ (transforming growth factor-β) isoforms and different chromatin accessibility.

## 1 Introduction

The vertebrate retinae are a structured neural tissue composed of retinal neurons, retinal glial cells, and retinal pigment epithelial cells. Retinal neurons form multiple circuits to produce a visual output,^1^ while retinal pigment epithelial cells and glial cells contribute to maintaining the integrity and homeostasis of the retinae and supporting retinal nerve cells.^2^ The irreversible loss of retinal nerve cells, mainly the retinal ganglion cells and the photoreceptors, causes permanent blindness.^3^

Retinal Müller glial cells (RMGCs) are the predominant glial cells in the retinae. They are found in the inner nuclear layer (INL) and span all retinal layers.^4^ In a healthy retina, RMGCs contribute to maintaining the normal structure and function of the retinae by establishing the retina–blood barrier, mediating the transport of ions and water, and providing nutritional and antioxidant support for retinal cells.^5^^,^^6^ In an impaired retina, various neurotrophic and growth factors secreted by RMGCs, such as the fibroblast growth factor (FGF) and ciliary neurotrophic factor (CNTF), protect of the retinal cells from injury.^7^^,^^8^ However, RMGCs respond differently to injury in different species.

**Fig. 1:**
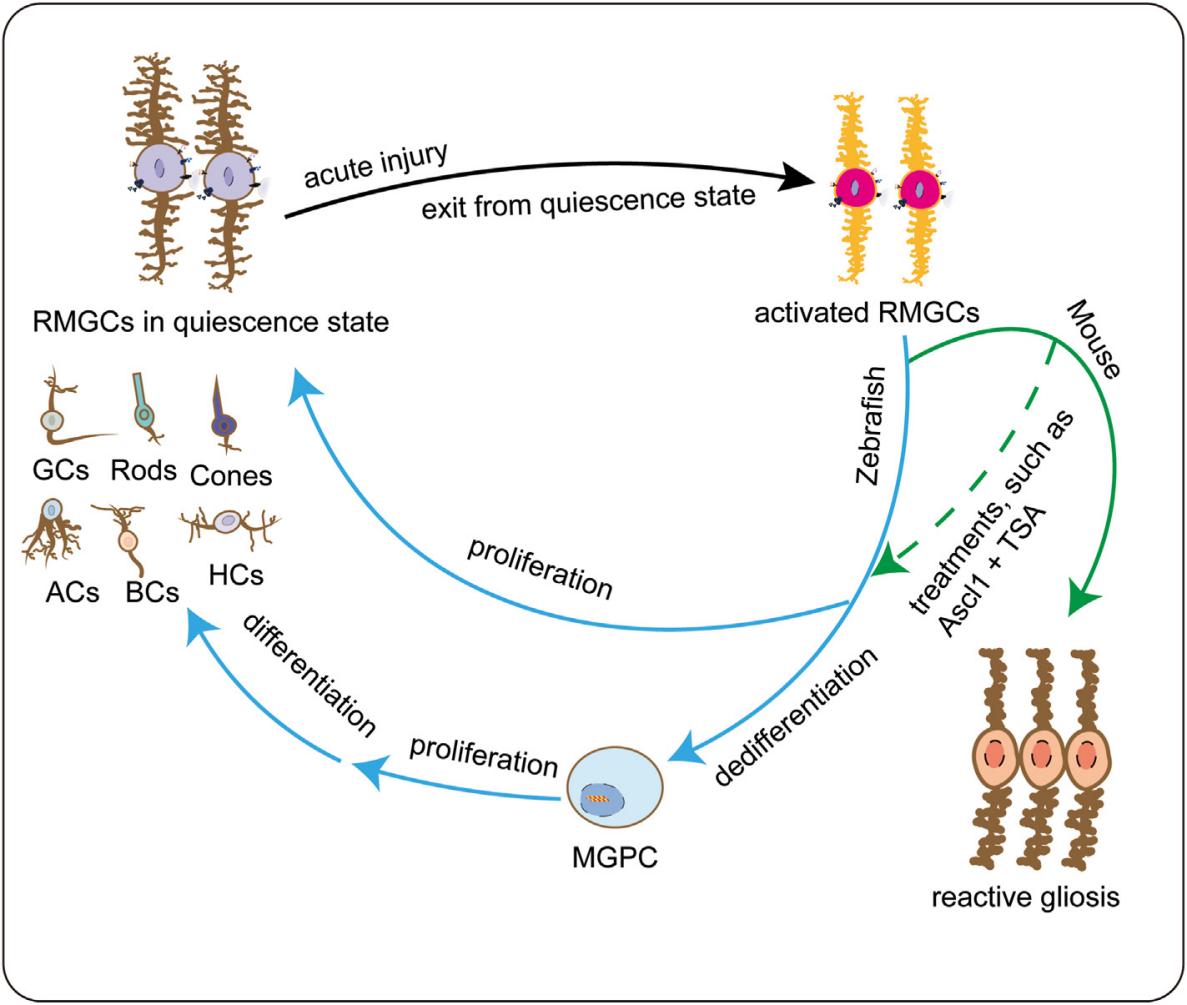
The response of retinal Müller glial cells upon acute injury in zebrafish and mouse reitnae. In zebrafish, the retinal damage spontaneously triggers RMGC reprogramming. The reprogramming process include the exit from quiescence state, the dedifferentiation of RMGCs, and the proliferation and differentiation of MGPCs. In contrast, the RMGC undergoes reactive gliosis in injured mice retinae. Forced activation of RMGCs proliferation, such as treated with AsclI and TSA, can stimulate the RMGC reprogramming in mice retinae. Abbreviations: RMGCs retinal Müller glial cells; MGPCs Müller glia-derived progenitors; ACs Amacrine cells; GCs Ganglion cells; HCs Horizon cells; BCs bipolar cells; TSA Trichostatin A.

In teleost fish, such as zebrafish, RMGCs play a crucial role in the spontaneous recovery of injured retinae.^9^^,^^10^ They undergo cell reprogramming events to produce Müller glia-derived progenitor cells (MGPCs), which further differentiate and proliferate to generate new retinal cells for repairing injuries (Fig. 1). Zebrafish RMGCs can respond to various forms of injury, and all types of retinal nerve cells can be regenerated through RMGC reprogramming.^4^ Similarly, postnatal chicks exhibit a limited neural regeneration responding to retinal injury through RMGCs proliferation and regenerate retinal nerve cells.^11^^,^^12^ However, in mammals, the main response of RMGC to a retinal injury is reactive gliosis, which forms a physical barrier to protect the tissue from further damage.^13^^,^^14^ Unfortunately, reactive gliosis leads to the formation of glial scars and inhibits the regeneration of damaged retinal tissue.^15^^,^^16^ Although reactive gliosis also occurs in teleost fish when neuronal death occurs, permanent suppression of regeneration does not happen.^17^^,^^18^

It has been uncovered that a number of genetic and epigenetic regulators play a crucial role in stimulating the reprogramming of RMGCs. Treated with certain regulators, mammalian RMGCs demonstrate the ability to regenerate retinal neurons.^19^, ^20^, ^21^ In this review, we summarize these important regulators that are involved in the RMGC reprogramming process and highlight their activity in different animal models.

## 2 Signalings initiating RMGC reprogramming

**Fig. 2:**
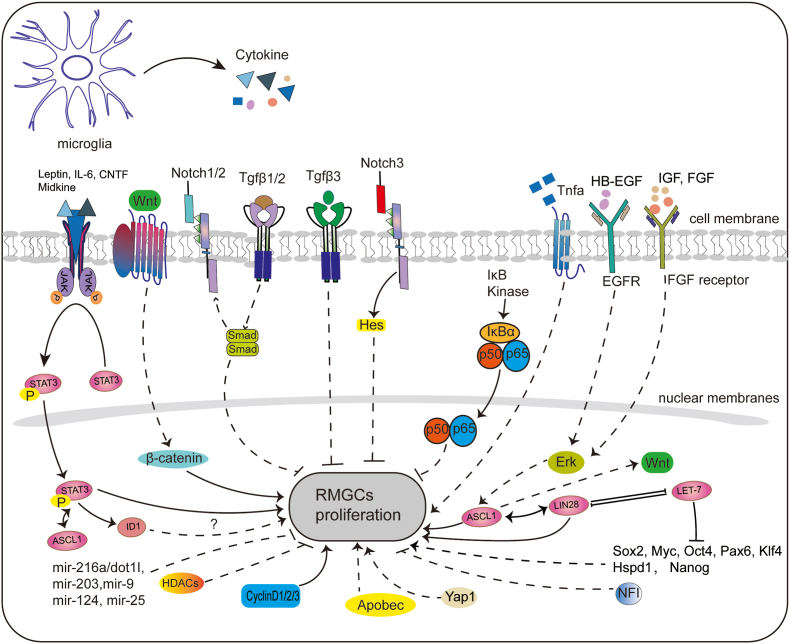
The genetic and epigenetic regulatory networks involved in retinal Müller glial cell reprogramming. Abbreviations: RMGCs retinal Müller glial cells.

In lower vertebrates, RMGCs sense injury and initiate reprogramming through various growth factors or signaling pathways. Current researchers have identified several important signalings, including Notch and TGFβ signalings, cytokines, growth and neurotrophic factors (Fig. 2).

RMGCs support retinal homeostasis at a quiescent state in a healthy retina. Hoang and his colleagues have reported that RMGCs transiently exits from the quiescent state after injury in all three species (zebrafish, chick and mice). RMGCs in zebrafish and chick pass through this reactive state, however, RMGCs in mice rapidly return to quiescence.^22^ Notch and TGFβ signalings are required for the maintenance of the quiescence state of RMGCs in zebrafish retinae.^12^^,^^17^^,^^23^, ^24^, ^25^, ^26^, ^27^, ^28^, ^29^, ^30^, ^31^, ^32^, ^33^, ^34^, ^35^, ^36^

Diverse Signaling by TGFβ isoforms exhibit different function in response to retinal injury. The activation of TGFβ1 and TGFβ2 leads to retinal gliosis, while TGFβ3 regulates retinal regeneration.^17^^,^^23^^,^^26^^,^^27^ In response to injury, Tgfβ1b and 2 are upregulated in zebrafish RMGCs, but are subsequently downregulated, while the TGFβ3 is initially downregulated and maintains a certain level throughout the process.^23^^,^^25^^,^^26^ Overexpression of TGFβ3 inhibits injury-dependent RMGCs proliferation, while inhibition of TGFβ3 signaling induces increased RMGCs proliferation.^23^, ^24^, ^25^ Interestingly, TGFβ3 signaling is not active in mice.^19^ Tgfβ1b overexpression leads to retinal gliosis without affecting RMGCs proliferation in injured retinae, probably through the p38 ​MAPK signaling pathway.^23^^,^^26^

Similarly, in zebrafish, Notch3 is expressed in the undamaged retinae and is downregulated in response to the retinal damage.^30^ The repression of Notch signaling activates the proliferation of RMGCs, through promoting the expression of regeneration-associated genes, including *stat3, hb-egf, mycb, socs3b,* and *ascl1a*.^29^, ^30^, ^31^ In the avian retinae, Notch signaling is upregulated to promote the RMGCs dedifferentiation and proliferation in the lesioned retinae, while later it inhibits the differentiation of the newly generated progenitor cells.^33^ In mammals, *Notch1/*2 ​mRNA is upregulated after laser focal injury, leading to the formation of a glial scar.^17^ It is worth noting that inhibition of Notch signaling rescues RMGCs proliferation in TGFβ3 overexpressing zebrafish, suggesting a collaboration between these two signaling pathways during RMGC reprogramming, although these signalings can also act independently in RMGC reprogramming.^23^^,^^34^ NFI factors play an important role in restoring RMGCs quiescent state in mice. NFI factors is upregulated after retinal injury. Deletion of NFI factors a, b, and x resulted in Müller glial cell reprogramming into retinal neurons in adult mice after injury.^22^

Microglia are the resident macrophages in retina, the widely accepted player in inflammation. When sensing the injury, microglia rapidly migrate to the site of cell death and undergo pronounced changes in morphology and gene expression pattern.^37^ Activated retinal microglia is essential for RMGC reprogramming through interacting with RMGCs and releasing cytokines and chemokines in zebrafish.^38^^,^^39^ The depletion of retinal microglia induced with pharmacological treatments hinders retinal regeneration in injured zebrafish retina.^38^^,^^40^, ^41^, ^42^ Different from in zebrafish, microglia-mediated inflammation promotes mammalian Müller glial reactive gliosis in response to injury.^14^^,^^16^^,^^38^^,^^43^, ^44^, ^45^ Numerous cytokines and chemokines are upregulated in activated retinal microglia and RMGCs in response to retinal injury.^22^^,^^46^ Cytokines, including Nuclear Factor Kappa B (NF-κB), Tumor necrosis factor-alpha (TNFα), interleukin family members and leptin, have been identified as the important activators for RMGCs proliferation.^23^^,^^29^, ^30^, ^31^, ^32^, ^33^^,^^35^

NF-κB is a key regulator of inflammation. NF-κB signaling is activated in RMGCs in injured mice retinae depending on microglia. The elimination of microglia by PLX5622 inhibit the activation of NF-κB signaling.^45^ Active NF-κB signaling plays an important role as a signaling hub in restoring the resting state,^22^ initiating the gliosis,^45^ and suppressing the reprograming of RMGCs in mice and chicks.^47^^,^^48^ Inhibition of NF-κB in injured retinae significantly enhances the reprogramming of RMGCs into neuron-like cells.^47^^,^^48^ A comparative transcriptome analysis suggests NF-κB pathway is upregulated in response to injury in zebrafish,^46^ however, the role of NF-κB in the reprogramming of RMGCs in zebrafish is not reported yet.

TNFα is initially released from dying retinal neurons and required for RMGCs proliferation through promoting the Stat3 and Ascl1a expression in RMGCs.^12^^,^^49^ Co-injection of Notch signaling inhibitor RO4929097 and TNFα induces RMGC reprogramming into neurons in the undamaged zebrafish retina.^29^ In cultured RMGCs derived from light-damaged mouse retinae, the elevation of TNFα promotes RMGCs proliferation. However, the proliferated RMGCs do not enter the reprogramming process to generate MGPCs and new neurons, but instead contribute to Müller glial reactive gliosis.^36^^,^^50^ TNFα signaling and NF-κB signaling mutually reinforce in Müller glial reactive gliosis in mice.^48^^,^^51^^,^^52^ In the human retinal organoids, combined application of TNF and HB-EGF induces photoreceptor degeneration, glial pathology, laminar disorganization, and scar formation.^53^

Leptin and IL-6 family cytokines (such as IL-6 and CNTF) have also been reported to synergize to stimulate RMGCs proliferation via Jak/Stat3 and Mapk/Erk signaling pathways in the uninjured and injured zebrafish retinae.^54^^,^^55^ Knockdown of CNTF receptor reduces RMGCs proliferation in light-damaged zebrafish retina.^56^ However, Fischer and his colleagues reported that intraocular injection of BMP4 and CNTF leads to the opposite result in the injured chicken retinae.^57^ In mammals, CNTF binds to the gp130 receptor, activating the Jak/Stat3 signaling pathway in RMGCs and regulating GFAP levels, leading to the character of Müller glial reactive gliosis.^58^, ^59^, ^60^

Secreted growth factors and neurotrophic factors, including Insulin, IGF1, HB-EGF, FGF, and Midkine, have also been reported to play important roles in activating RMGC reprogramming. HB-EGF and Insulin are sufficient to stimulate the formation of multipotent progenitors in zebrafish retinae with certain injury.^49^^,^^61^^,^^62^ These growth factors may synergize with each other to more effectively promote RMGCs proliferation via the Mapk/Erk and Jak/Stat3 signalings.^61^^,^^62^ Furthermore, the mRNAs of *hb-egfa*, *insulin* and *igf1* are upregulated at the site of injury in zebrafish retinae. Similarly, in the postnatal chick and rodent retinae, RMGCs proliferation can be induced though HB-EGF with retinal damage, the combination of FGF2 and Insulin, or the combination of IGF1 and FGF2, although most of the newly formed cells may be progenitor-like cells but not retinal neurons.^63^, ^64^, ^65^ Insulin and bFGF also play a role in guiding the migration of RMGCs in response to neuronal loss in mice retinae.^66^ Midkine is another growth factor which may play a critical role in RMGC reprogramming. Injury induces a rapid upregulation of *midkine* in zebrafish and chicken, but a downregulation in mice RMGCs.^67^^,^^68^ In *midkine-a* mutant zebrafish, the proliferation of RMGCs was suspended. RMGCs undergo reactive gliosis in response to the photoreceptor death.^67^ Similar within zebrafish, inhibition of Midkine signaling increases cell death and reduces MGPCs formation in chick retinae.^68^

## 3 Regulators mediating the dedifferentiation and proliferation of RMGCs

Once initiated, RMGCs undergo dedifferentiation and proliferation. To date, a series of internal cell signalings, transcription factors, epigenetic factors, and microRNAs have been identified as important regulators in this process.

### 3.1 The internal cell signalings and transcription factors

The activation of multiple internal cell signalings and the changing of transcriptional patterns are necessary for the generation and proliferation of MGPCs when RMGCs exits from the quiescent state.

Activation of Jak/Stat3 and Mapk/Erk pathways by growth factors or cytokines appears to be responsible for the changing of transcriptional patterns of RMGCs. The intravitreal injections of HB-EGF and overexpression of Midkine activated Jak/Stat3 pathway, and the treatment with Insulin and Igf-1/FGF2 activated Mapk/Erk pathway.^61^^,^^62^^,^^67^ In zebrafish, phosphorylated stat3 (pStat3), the active form of Stat3, is only present in proliferating MGPCs, although *Stat3* is expressed in all RMGCs.^69^ Inhibition of Stat3 leads to a decrease in the number of proliferating MGPCs in the damaged retinae.^55^ The activation of Jak/Stat3 and Mapk/Erk signalings leads to the upregulation of transcriptional factors in MGPCs, such as *asclI*, a core transcriptional factor in MGPCs proliferation.^61^^,^^67^

Similar to zebrafish, the Jak/Stat3 and Mapk/Erk signaling pathway is activated in RMGCs in the NMDA-injured avian retinae, leading to the generation of MGPCs.^70^ The activation of Jak/Stat3 signaling even is sufficient to induce RMGC reprogramming in the absence of retinal damage.^70^ However, in mammals, although Jak/Stat3 signaling is also upregulated, the capacity for RMGC reprogramming and the expression of *Ascl1* is inhibited.^22^^,^^71^ The transcriptional regulator ID1 protein, one of the potential targets of Jak/Stat3 signaling,^19^ may be a key factor to mediating the difference between injured zebrafish and mammalian retinae. In zebrafish, *id1* is upregulated in response to injury.^72^ In mammals, *Id1* is induced early in injury but rapidly reverts to the basal level.^19^ ID1 has been reported are regulator of several genes such as *hes, tcf3* and *neuroD*.^72^ Further evidence is required to substantiate the role of ID1 in MGPCs proliferation. Furthermore, activation of Jak/STAT3 signaling induces NF-κB p65 translocation from the cytoplasm into the nucleus, resulting in Müller glial reactive gliosis in mice.^51^^,^^73^ These studies suggest that Stat3 may be a key regulator of early MGPCs proliferation.

Recently, studies have suggested that the induction of Ascl1a/Lin28/Let-7signaling is a critical event in the RMGCs proliferation and dedifferentiation. Ascl1a is a basic-helix-loop-helix (bHLH) transcription factor firstly described as pro-neural factor in the context of neural differentiation.^74^ Lin28 is an RNA binding protein with an important role in cell growth through modulation of the expression of cell cycle genes *cyclins* and microRNA *let-7*.^75^^,^^76^ MicroRNA *Let-7* represses the expression of *ascl1a, hspd1, lin28, oct4, pax6b* and *myc*.^77^ Ascl1a activates the expression of*lin-28*. Clustered LIN28 in RMGCs inhibits the expression of microRNA *Let-7*.^77^ In zebrafish, injury leads to a rapid upregulation of *ascl1a* and *lin28*in mitotic MGPCs, and Ascl1a and Lin-28 knockdown inhibit MGPCs proliferation.^77^ Apart from its role in MGPCs proliferation, Ascl1a can regulate the initiating signalings of RMGC reprogramming, such as *stat3, wnt4a* and *notch3*.^78^

Differing from zebrafish, *Ascl1* is not expressed after retinal injury in mammals.^79^ Interestingly, the overexpression of *Ascl1* can induce RMGCs’ reentry into the cell cycle in vitro and trigger the production of various retinal nerve cells.^80^ Furthermore, the forced expression of *Ascl1* initiates an injury response that stimulates retinal regeneration in young mice, in which RMGCs give rise to amacrine and bipolar cells as well as photoreceptors.^79^ Moreover, the combination of Ascl1 overexpression and the histone deacetylases (HDACs) inhibitor trichostatin A (TSA) treatment can induce RMGC reprogramming and differentiation into amacrine and bipolar cells in adult mice.^20^ Inhibition of NF-κB significantly promotes the RMGC reprogramming stimulated by overexpression of AsclI and TSA treatment.^45^ Similarly, combined application of AsclI and additional transcriptional factors, such as pro-neural factors Atoh7, Pou4f2, Otx2 and Islet1, remarkably promotes the regenerative capacity of RMGCs in mice retinae, although the application of additional transcriptional factors may affect the cell fate decision of MGPCs.^81^, ^82^, ^83^ These findings suggest that Ascl1 is a key factor in RMGC reprogramming, and epigenetic regulators may facilitate the function of Ascl1 by providing a favorable microenvironment.

In recent years, researchers have reported that the common factors of cell proliferation and stemness, such as Hippo pathway, mTOR pathway, PTEN-PI3K/AKT pathway, Wnt/β-Catenin Pathway, Hedgehog Pathway and pluripotent transcription factors are involved in MGPCs proliferation.^36^^,^^84^, ^85^, ^86^, ^87^, ^88^, ^89^ Lourenço and his colleagues have reported that the Hippo pathway facilitates RMGCs to exiting the resting state and reenter the cell cycle by stimulating Ascl1a/Lin28/Let-7 signaling in RMGCs.^84^ YAP1 is quickly upregulated in injured retinae. Knockdown of *yap1* in injured retinae leads to a decrease in the MGPCs proliferation.^22^ YAP1 is also required for MGPCs proliferation in Xenopus.^85^ Similarly in zebrafish, forced *yap1* expression in the mammalian retinae stimulates RMGCs proliferation and upregulates *cyclinD1* and *cyclinD3* mRNA levels both in Vitro and in Vivo.^85^^,^^86^ Mice treated with NMDA exhibit an increased level of pYAP/YAP.^86^ Consistently, Kastan N and his colleagues have also showed that the small molecule inhibitors of Lats kinases induce RMGCs proliferation in human retinal organoids.^90^^,^^91^ Zhang and his colleagues have demonstrated that mTOR is rapidly activated by microglia/macrophage-mediated retina inflammation in RMGCs and MGPCs following injury in zebrafish, the inhibition of mTOR suppresses the dedifferentiation of RMGCs and the proliferation MGPCs.^37^^,^^92^ In addition, Gupta and his colleagues have reported that downregulation of Pten activates Akt and enhances MGPCs proliferation in zebrafish.^93^ Gao and his colleagues have reviewed the current knowledge regarding the roles of Wnt/β-Catenin and Hedgehog Pathway in RMGC reprogramming.^36^^,^^94^^,^^95^

Multiple pluripotent transcription factors, such as Sox2, Sox11, Oct4, Myc, and Klf4 are upregulated and expressed in MGPCs following retinal injury, and their expression is regulated by Ascl1a/Lin28/Let-7 signaling.^87^, ^88^, ^89^^,^^96^ In zebrafish, gene knockdown and overexpression showed Sox2 is necessary and sufficient for Müller glia proliferation.^87^ Oct4 and Myc are closely associated with epigenetic factors during the exit of the cell cycle.^88^^,^^89^ Sox11b regulates the migration and differentiation of MGPCs during retinal regeneration in zebrafish.^96^ However, the role of these factors play in mammalian RMGCs is not clear. Previously, a single Sox2 is proved to reprogram astrocytes into proliferative neuroblasts in the adult mouse brain.^97^ Recently, ectopic expression of the *Oct, Sox2* and *Klf4* genes stimulated retinal ganglion cell reprogramming and promoted axon regeneration after injury by restoring youthful DNA methylation patterns and transcriptomes.^21^ These studies suggest the necessity of the expression of multiple pluripotent transcription factors, which may be closely related with epigenetic modification.

### 3.2 The epigenetic factors

Epigenetic modifications also play a role in inducing RMGC reprogramming. The activation of RMGCs requires a more open and accessible state of chromatin. Chromatin accessibility after injury displays species-specific changes.^98^ For instance, the *ascl1a* promoter became more accessible after injury in zebrafish but not in mice.^22^ The overexpression of Oct4, Sox2, and Klf4 (OSK) in retinal ganglion cells restores youthful DNA methylation patterns and promotes axon regeneration in injured or aged mice.^21^^,^^99^

Powell and his colleagues reported that many of DNA demethylation regulators were induced in the injured retinae, such as *gadd45β, gadd45βl, gadd45γ, gadd45γl, apobec2a, apobec2b, dnmt,* and *tet3*.^100^ Treatment of 5-aza-2′-deoxycytidine in injured zebrafish induced global DNA hypomethylation and upregulated the expression of *tubulin1a,*suggesting the formation of MGPCs.^101^ In addition, differentially methylated bases analysis (DMB) methylation showed a correlation between promoter DMBs with decreasing methylation and increased gene expression. Pluripotency factor and regeneration-associated genes, such as *oct4, klf4, sox2, c-myc, lin28,* and *nanog*, are hypomethylated in quiescent RMGCs until MGPCs.^101^ Interestingly, similar to zebrafish, pluripotency factors and genes associated with regeneration had low methylation levels in isolated mouse RMGCs from uninjured retinae.^101^ The pluripotency-related gene *Oct4* is expressed after injury but is quickly silenced by methylation.^102^ Moreover, *Apobec1* (promotes the DNA demethylation) is upregulated in the retinae treated with NMDA and EGF, promoting *Nestin* expression in cultured RMGCs and facilitating RMGCs dedifferentiation.^103^ This evidence suggests that injury-induced RMGC reprogramming in mammals may be limited by DNA methylation.

In addition to DNA methylation, other epigenetic modifications, such as histone modification is equally important. Histone deacetylases (HDACs) are a class of vital epigenetic factor that plays significant roles in cellular homeostasis and tissue differentiation.^104^ In zebrafish, the levels of *hdac3, hdac5,* and *hdac6* mRNA increased early after injury. The blocking of HDACs by valproic acid (VPA) suppresses the proliferation of MGPCs and downregulates genes *lin28* and*insm1a* accompanied by a decrease in acetylated histone H4.^105^^,^^106^ In mice, co-injection of TSA and STAT inhibitors effectively induces Müller glia-derived neurons.^19^ The forced expression of *Ascl1a* and the treatment of TSA also induce Müller glia to reprogram in adult mouse retinae.^20^ In TSA-treated mouse retinae, the level of histone H3K27 acetylation significantly increases. Recently, Campbell and his colleagues have demonstrated that S-adenosylhomocysteine hydrolase (SAHH) and histone methyltransferases (HMTs) are required for the formation of MGPCs in chick.^107^ These studies suggest that more open chromatin accessibility and progenitor-associated genes may also be key factors in Müller glial cell reprogramming.^20^

### 3.3 MicroRNA

MicroRNAs (miRNAs) are short, noncoding RNA molecules that regulate gene expression by binding to messenger RNA (mRNA) and inhibiting its translation into proteins.^108^^,^^109^ In zebrafish, Dicer is the main enzyme responsible for the formation of miRNAs. Knockdown of Dicer in the zebrafish eyeball using intravitreal injection of MO has been shown to hinder the reentry of RMGCs into the cell cycle,^110^ suggesting that miRNAs play a crucial role in controlling the early stages of regeneration.

MicroRNA Let-7, a highly conserved histiocyte-specific miRNA, has been found to inhibit the expression of regeneration-related genes such as *ascl1a, hspd1, oct4, pax6b,* and *lin28,* thus regulating the proliferation of Müller glia.^77^^,^^111^ In zebrafish, microRNA mir216a has been found to directly target and degrade the H3K79 methyltransferase Dot1l leading to Müller glia proliferation through the *wnt/β-catenin* signaling pathway.^94^ In mammals, previous studies have also shown that miR-9 and miR-124 can facilitate Ascl1-induced RMGCs proliferation in primary cultures.^112^ Recent research has found that knockdown of the RNA-binding protein PTBP1 can lead to the proliferation of RMGCs and convert them into functional retinal ganglion cells.^113^ PTBP1 is a necessary regulator of mRNA translation and is controlled by miR-9 and miR-124.^114^, ^115^, ^116^

Moreover, miRNAs have also been found to regulate the proliferation of MGPCs in addition to their role in MGPCs formation. Sequencing results have shown that miR-7a, miR-2142b, miR-2146a, miR-27C, and miR-231 play a role in progenitor cell proliferation and migration.^110^ miR-203 has been found to negatively regulate the proliferation of MGPCs by inhibiting Pax6b.^117^ In mouse retinae, in vitro experiments have shown that miR-7a can negatively regulate MGPCs differentiation by targeting the *Notch3* 3′-UTR motif,^118^ while anti-miR-28 can induce MGPCs to differentiate into neurons by targeting *crx*.^119^

## 4 Conclusions

In conclusion, comparative analysis shows that the major difference between mouse and zebrafish are the early response of RMGCs following retinal injury, such as the regulation in the exit from quiescent state, the initiation of reactive gliosis, and the re-entry of cell cycle of RMGCs. Once MGPCs was forcedly generated in injured mice retinae, for instance, through the co-overexpression of AsclI and pre-neural transcriptional factor Atoh7, RMGCs can be reprogrammed to generate new retinal neurons. Diverse regulation of Notch and TGFβ isoforms and different chromatin accessibility may be responsible for the different early response upon injury stimulation between mouse and zebrafish.

Although a number of factors have been identified, there is still a long way to go before uncovering the dedicated regulatory networks in RMGC reprogramming. Numerous candidate factors and networks have been suggested through comparative omics studies, such as RNA-sequencing, single cell RNA-sequencing, transposase-accessible chromatin with high-throughput sequencing (ATAC- sequencing) analyses,^22^^,^^120^, ^121^, ^122^ more studies are expected to reveal their roles in RMGC reprogramming.

## Study Approval

Not Applicable.

## Author contributions

Conception and design of study: XX, ZL and JZ; Data collection: XX; Drafting the manuscript: XX, ZL and JZ; All authors reviewed the results and approved the final version of the manuscript.

## Funding

This research did not receive any specific grant from funding agencies in the public, commercial, or not-for-profit sectors.

## Declaration of competing interest

The authors declare that they have no known competing financial interests or personal relationships that could have appeared to influence the work reported in this paper.

## Abbreviations

RMGCs: Retinal Müller glial cells
INL: Inner nuclear layer
FGF: Fibroblast growth factor
CNTF: Ciliary neurotrophic factor
NF-κB: Nuclear Factor Kappa B
TGFβ: Transforming growth factor-β
HDACs: Histone deacetylases
TSA: inhibitor trichostatin A
MGPCs: Müller glia-derived progenitor cells
